# Life cycle assessment of a rainwater harvesting system compared with an AC condensate harvesting system

**DOI:** 10.1016/j.resconrec.2019.01.043

**Published:** 2019

**Authors:** Santosh R. Ghimire, John M. Johnston, Jay Garland, Ashley Edelen, Xin (Cissy) Ma, Michael Jahne

**Affiliations:** a Global Sustainability and Life Cycle Consultant, LLC, U.S. Environmental Protection Agency, 960 College Station Rd, Athens, GA, 30605, USA; b U.S. Environmental Protection Agency, Office of Research and Development, Computational Exposure Division, 960 College Station Rd, Athens, GA, 30605, USA; c U.S. Environmental Protection Agency, Office of Research and Development, 26 W. Martin Luther King Dr, Cincinnati, OH, 45268, USA; d Oak Ridge Institute for Science and Education Participant, US Environmental Protection Agency, 26 W. Martin Luther King Dr, Cincinnati, OH, 45268, USA

**Keywords:** Air-conditioning condensate water, Life cycle assessment, Rainwater harvesting, Water reuse

## Abstract

This study presents a life cycle assessment (LCA) of a rainwater harvesting (RWH) system and an air-conditioning condensate harvesting (ACH) system for non-potable water reuse. U.S. commercial buildings were reviewed to design rooftop RWH and ACH systems for one to multi-story buildings’ non-potable water demand. A life cycle inventory was compiled from the U.S. EPA’s database. Nine scenarios were analyzed, including baseline RWH system, ACH system, and combinations of the two systems adapted to 4-story and 19-story commercial buildings in San Francisco and a 4-story building in Washington, DC. Normalization of 11 life cycle impact assessment categories showed that RWH systems in 4-story buildings at both locations outperformed ACH systems (45–80% of ACH impacts) except equivalent in Evaporative Water Consumption. However, San Francisco’s ACH system in 19-story building outperformed the RWH system (51–83% of RWH impacts) due to the larger volume of ACH collection, except equivalent in Evaporative Water Consumption. For all three buildings, the combined system preformed equivalently to the better-performing option (≤4–8% impact difference compared to the maximum system). Sensitivity analysis of the volume of water supply and building occupancy showed impact-specific results. Local climatic conditions, rainfall, humidity, water collections and demands are important when designing building-scale RWH and ACH systems. LCA models are transferrable to other locations with variable climatic conditions for decision-making when developing and implementing on-site non-potable water systems.

## 1. Introduction

Globally, 71% of irrigated areas and 47% of large cities (> 500,000 inhabitants) are reported to experience periodic, annual, seasonal, or dry year water shortages (Brauman et al., 2016). Innovative water management and decentralized green infrastructure practices are emerging as strategies to address water resources sustainability issues worldwide (County, 2009; Ghimire and Johnston, 2015; Ghimire et al., 2017). Some examples are rainwater harvesting (RWH); atmospheric water harvesting that includes air-conditioning (AC) condensate water, atmospheric water generation from desert air with low relative humidity to 20% (Kim et al., 2017; Fathieh et al., 2018), and fog water collection; as well as on-site gray water treatment and reuse (USEPA, 2012; Schoen et al., 2015). Our research focuses on RWH and AC condensate harvesting, which have similar water quality and treatment requirements yet may be better suited for different building configurations and climate conditions.

Approximately 9% of fresh water in lakes and rivers on Earth (i.e., 12,900 km^3^ of 179,000 km^3^) takes the form of water vapor and droplets in the atmosphere (Gleick, 1993; Graham et al., 2010). Condensate is water collected on a cool surface such as that in the evaporator section of the air-handling unit (AHU) of a Heating, Ventilation, and Air-Conditioning (HVAC) System (Glawe, 2013). AC condensate and rainwater can be used for make-up water in cooling towers, in addition to other non-potable uses such as toilet and urinal flushing, irrigation, ornamental water features, and manufacturing processes (Glawe, 2013; Ghimire et al., 2014). Most cooling systems in U.S. buildings are Packaged Air Conditioning Units (37%) and Residential-Type Central Air Conditioners (30%) (EIA, 2016).

Literature has addressed many aspects of RWH assessment. A few examples include design and feasibility assessment (Eroksuz and Rahman, 2010; Mahmood and Hossain, 2017; Moniruzzaman and Imteaz, 2017); human health impacts (Domenech et al., 2012); performance and effectiveness (Ward et al., 2012; Zhang and Hu, 2014); importance of temporal lumping of rainfall data (Mitchell, 2007; Mitchell et al., 2008); energy implications (Siems and Sahin, 2016); hydrologic impacts (Glendenning and Vervoort, 2011; Welderufael et al., 2011; Ghimire and Johnston, 2013; Shuster and Rhea, 2013; Walsh et al., 2014); life cycle costs and cost-efficiency (Farreny et al., 2011; Roebuck et al., 2011; Ghimire et al., 2012); and life cycle assessments (Ghimire et al., 2014; Wang and Zimmerman, 2015; Ghimire and Johnston, 2017) of RWH. More recently a life cycle assessment (LCA) of a commercial RWH system was performed and compared to a municipal water supply system adapted to Washington, D.C, and reported that the benchmark RWH system outperformed the municipal water supply system in all categories except Ozone Depletion (Ghimire et al., 2017). In a prior study, Ghimire et al. (2014) performed LCA of domestic and agricultural RWH systems adapted to the Southeast U.S. and reported that minimal RWH designs with no pumps reduced environmental impact from 78% energy use to 88% human health criteria pollutants.

LCA is widely accepted tool used in diverse sectors to assess environmental and human health impacts in a cradle-to-grave approach consistent with the International Organization for Standardization (ISO) guidelines (ISO, 2006b, a). Relatively less literature exists on assessing AC condensate collection rates (Painter, 2009; Lawrence et al., 2010; Licina and Sekhar, 2012; Cook et al., 2014) and even less attention has been paid to life cycle feasibility and life cycle environmental implications of AC condensate harvesting combined with RWH. In addition, wider adoption of these innovative green infrastructure practices is dependent on availability of data and analyses to support their effective use. Therefore, this study focuses on life cycle impacts assessment of the RWH and ACH systems.

### 1.1. Objectives, scope, and novelty

The main objective of this study was to conduct LCA of RWH and AC condensate harvesting (ACH) systems, separately and in combination. Nine scenarios of RWH and ACH were addressed. Three systems, namely, baseline RWH system, ACH system, and the combined RWH and ACH systems were adapted to one 4-story and one 19-story commercial building in San Francisco, California (CA) and one 4-story building in Washington, District of Columbia (DC). Although the suburban DC area might have taller buildings, DC in particular may not have 19-story buildings and were excluded from the analysis. To our knowledge, no previous study has simultaneously addressed LCA of RWH and ACH systems. Specific objectives were to compile appropriate life cycle inventory (LCI) data, create models for LCA, and perform life cycle impact assessment (LCIA). Eleven LCIA category indicators: Acidification; Cumulative Energy Demand; Eutrophication; CO_2_ Emission; Fossil Depletion; Freshwater Withdrawal; Human Health Criteria (particulate matter equivalent); Metal Depletion; Ozone Depletion; Smog; and Evaporative Water Consumption per functional unit of 1 m^3^ of RWH and ACH delivery for flushing toilets and urinals were assessed, consistent with Ghimire et al. (2017).

A wide collaboration among experts on LCA, AC condensate, and RWH provided realistic and transparent data making this work scalable to other locations. This study complements U.S. EPA Office of Research and Development (ORD) parallel efforts on sustainability analysis of stormwater management practices and integrated assessment of decentralized non-potable water systems. ORD is currently evaluating the feasibility and sustainability (life cycle cost and environmental impacts/benefits) of green infrastructure practices and innovative non-potable water reuse configurations to enable well-informed decisions regarding sustainable water management. The following sections describe our approach, including tools, databases, assumptions and results, with concluding remarks on potential implications.

## 2. Approach

**Fig. 1.**
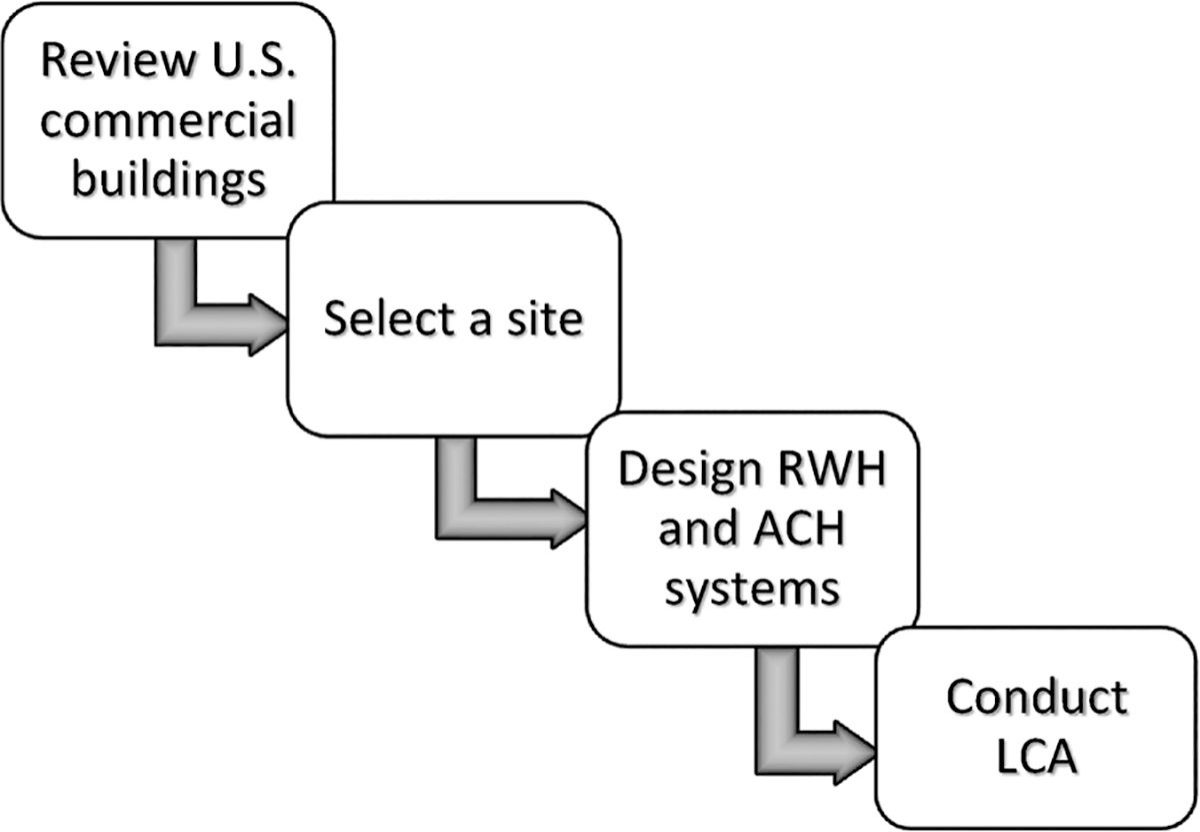
A four-step workflow diagram for LCA of RWH and ACH systems.

**Fig. 2.**
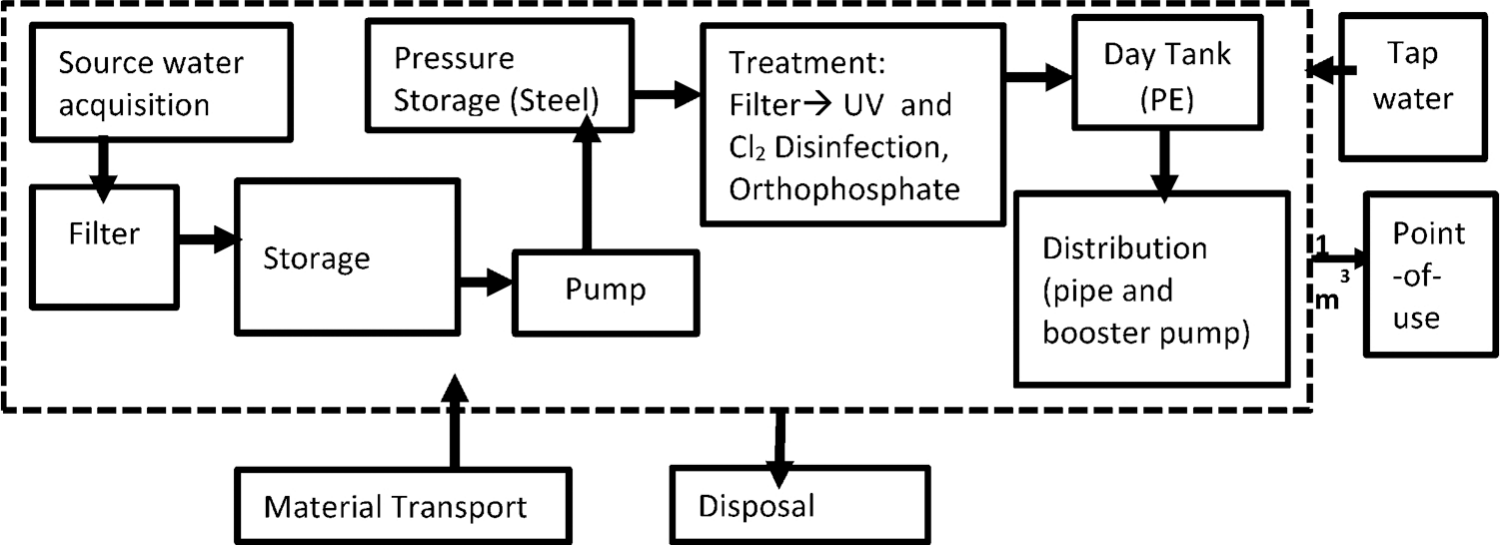
LCA system boundary of a RWH, compared to an ACH system. The source water for RWH and ACH systems includes rain water and vapor condensation, respectively (figure modified from Ghimire et al. (2014) and Ghimire et al. (2017)). The LCA system boundary spans cradle-to-grave, excluding the distribution of both systems’ components from final manufacture to point of use and disposal phases, consistent with Ghimire et al. (2017).

A general approach for conducting LCA of RWH and ACH systems is depicted in Fig. 1, with the system boundary shown in Fig. 2. The U.S. commercial buildings were first reviewed, the building sites were then selected, and the systems were designed for the selected sites. The designs provided the necessary input parameters for the LCA calculations.

### 2.1. Review U.S. commercial buildings

Public use microdata of the Commercial Buildings Energy Consumption Survey 2012 (CBECS), published online by the U.S. Energy Information Administration (EIA, 2016), were reviewed to obtain data related to U.S. commercial buildings. The microdata represents survey data representing commercial buildings from the 50 States and the District of Columbia. Specific data reviewed were: number of commercial buildings; total floorspace (i.e., area); and number of floors, by Census Regions (Northeast, Midwest, South, and West). In addition, average building floorspace was estimated using total floorspace and number of buildings; average roof area was estimated using building floorspace and number of stories. The survey contact person at the Energy Information Administration was consulted to verify the building data completeness. It was found that the number of floors, i.e., the exact floor counts, were available up to a maximum of 14 floors; the floor information was grouped together for 15 to 25 floors and over 25 floors to maintain confidentiality of the building respondents.

### 2.2. Select a site

San Francisco, CA (West U.S. Census Region) and Washington, DC (South U.S. Census Region) were chosen for their readily available data, and EPA’s parallel studies on integrated assessment of decentralized non-potable water systems in these areas. Regional differences were considered by including local weather data (rainfall; temperature; psychrometric data of humidity ratio (moisture content); and dry air density) and regional building characteristics (building roof area; air conditioning floorspace; cooling capacity; and number of occupants.)

### 2.3. Design RWH and ACH systems

Baseline RWH and ACH systems were designed for flushing toilets and urinals in a 4-story commercial building serving 1000 occupants in San Francisco, CA by customizing a previously-published commercial RWH system in Washington, D.C. (Ghimire et al., 2017) (Table 1). Average monthly water demand and water collection rates of rainwater and AC condensate were calculated; and the minimum of the two (i.e., average monthly collection and demand) was selected to determine the storage tank. We note that estimating rainwater collection and storage tank size using gross average estimates of rainfall (e.g., monthly) are usually precise enough for design purposes (Glawe, 2013); however, other temporal lumping (daily or hourly rainfall data) may be used (Mitchell, 2007; Ghimire et al., 2012, 2017).

#### 2.3.1. Calculation of monthly water demand

Average monthly water demand was determined by calculating total annual water demand, *D*_*a*_, consistent with Ghimire et al. (2017):

(1) $$D_{a}\;=\;S_{n}\text{ x }N_{d}\text{ x }\left[Q_{t}\text{ x }\left(N_{f}\text{ x }F_{f}\;+\;N_{m}\text{ x }F_{m}\right)\;+\;Q_{u}\text{ x }N_{m}\text{ x }F_{u}\right]$$

where, *D*_*a*_ = Total annual water demand for flushing all urinals and toilets for a commercial building (liter/year = l/y) or (gallons/y = gal/y) *S*_*n*_ *=* Number of stories (floors) in a building *N*_*d*_ = Total number of days of system operation in a year (260 days/y) *Q*_*t*_ = Flush volume for low-flow toilets (4.8 l/flush or 1.28 gal/flush) *N*_*f*_ = Number of toilet-only users per floor (125) *F*_*f*_ = Number of toilet flushes per toilet-only user per day (3 flush/day, no urinals) *N*_*m*_ = Number of users using both toilet and urinal per floor (125) *F m* = Number of toilet flushes per user using both toilet and urinal per day (1 flush/day) *Q*_*u*_ = Flush volume for high-efficiency urinal demand (0.47 l/flush or 0.125 gal/flush) *F*_*u*_ = Number of urinal flushes per user per day (2 flush/day)

#### 2.3.2. Calculation of RWH collection rate and system design

An RWH storage tank is typically designed to store rainwater, using a drought factor of one-fourth annual demand (TXWDB, 2005) which can result in a larger, ineffective storage tank and greater initial investment cost. For purposes of the current study, the size of RWH storage tank was sized by comparing average monthly demand with average monthly collection volume (12-month averages). The storage tank was determined by choosing the minimum of the two monthly averages, i.e., collection and demand. To calculate the monthly rainwater collection, a 30-year mean monthly precipitation (January 1986 to December 2015) for San Francisco was obtained from NCEI (2018); the Washington, DC system is described by Ghimire et al. (2017). The 30-year mean annual precipitation for the site was 22.2″ or 0.56 m (Appendix Table A1). A spreadsheet model assessed average monthly water budget, a comparison of volumetric collection to demand, and size of RWH storage tank (TXWDB, 2005; Ghimire et al., 2014):

(2) $$V_{r}\;=\;c\text{ x }e\text{ x }p\text{ x }A$$

where *Vr=* RWH collection volume for each month (m^3^/month or gallons/month) *c =* Unit conversion factor (1.0 SI or 0.62 US Customary) *e =* Collection efficiency (0.75) (varies 0.75 to 0.90) (TXWDB, 2005) *p =* Precipitation (m/month or in/month), obtained from (NCEI, 2018) *A* = Roof area (m^2^ or ft^2^), obtained from (EIA, 2016)

Other components such as pipes (50.8 mm or 2 in polyvinyl chloride (PVC) and 38.1 mm or 1.5 in chlorinated PVC), filters, day tank and pressure tank, pumping energy (0.19 kW h/m^3^), and treatment (bag filter and UV) were consistent with Ghimire et al. (2017). Additional treatment processes of chlorination and corrosion inhibitor (orthophosphate) were included as best management practices for non-potable use, as proposed by the Water Environment & Reuse Foundation (Sharvelle et al., 2017).

The DC’s RWH storage tank size (76 m^3^) and % demand met (77%) were obtained from Ghimire et al. (2017).

#### 2.3.3. Calculation of ACH collection rate and ACH system design

The approach of fundamental mass balance between amount of water carried by the air entering and exiting the AHU, as previously used (Painter, 2009; Lawrence et al., 2010; Licina and Sekhar, 2012; Glawe, 2013; Cook et al., 2014), was applied to estimate the AC condensate collection rate. A spreadsheet model was developed for monthly average condensate collection volume, *V*_*c*_ (Eq. 3), by adopting the fundamental mass balance approach (Eq. 4).

(3) $$V_{c}\;=\;1,440\text{ x }D\text{ x }Q_{c}$$

(4) $$Q_{c}\;=\;k\text{ x }A_{e}\text{ x }Q_{a}\text{ x }\rho_{e}\text{ x }O_{a}\text{ x }\Delta H_{r}$$

where, *V*_*c*_ *=* Monthly condensate volume (m^3^/month or gal/month) *D* = Number of days in a month 1,440 = Unit conversion factor (i.e., 1 day = 1,440 min) ***Q***_***c***_ = Condensate production rate, liter per minute (lpm) or gal per minute (gpm) *k* = Weight of water factor (1.0 l/kg or 1/8.3 gal/lb) *A*_*e*_ = AHU operation efficiency (0–1, described below) *Q*_*a*_ = Airflow rate through the AHU, cubic meter per minute (cmm) or cubic feet per minute (cfm):

(4a) $$Q_{a}\;=\;C_{t}\text{ x }Q_{b}$$

*C*_*t*_ = AHU cooling capacity (tons) *Q*_*b*_ = Base airflow rates (cmm/ton) or (cfm/ton) *ρ*_*a*_ = Density of dry air (kg/m^3^) or (lb/ft^3^) *O*_*a*_ = Percentage of outside air entering the AHU (0–1) *ΔHr =* Difference in moisture content or humidity ratio (HR), water mass/dry air mass (kg/kg) or (lb/lb), between the incoming outdoor air and supply air leaving the AHU (varies with temperature)

In Eqs. 3 and 4, psychrometric data of humidity ratio, *ΔHr* (moisture content) and dry air density, *ρ*_*a*_, was obtained from Refrigeration Service Engineers Society’s Service Application Manual (RSES, 2009)

based on daily temperatures obtained from National Oceanic and Atmospheric Administration or NOAA (NCEI, 2018).

San Antonio Water System (SAWS) (Glawe, 2013) method and rule-of-thumb calculations, as reported at 3 to 10 gallons/day from a 1000 square feet of air-conditioned space (AWE, 2018), were also used to check ACH collection calculations; however, they are not included here.

A few assumptions were made to be consistent with peer literature:

- AHU operation efficiency,*A* *e*, was assumed at 0.25, consistent with a nominal 25% of total capacity in San Antonio, TX (Glawe, 2013) Airflow rate through the AHU, *Q*_*a*_ = *C*_*t*_ x *Q*_*b*_, was estimated using the following assumptions:
  - Cooling capacity, *C*_*t*_, was estimated as proportional to air-conditioning space, using the annual electric energy intensity (kWh/m^2^) for 100% floorspace cooled, as obtained from EIA’s conditional energy intensity (kWh/m^2^) by Census region, 2012.
  - Each floor was assigned an AHU that operates 64 h per week (52 weeks per year) (EIA, 2016).
  - For example: for San Francisco’s 4-story building, total cooling capacity, $C_{t}\;=\;\frac{187kWh}{m^{2}}x\;7,283m^{2}x\;\frac{1}{260x24h}x\;\frac{1\;ton\;}{3.52kW}\;=\;116\text{ tons}$ (refrigeration); this value was 85 tons for Washington, DC due to differences in floorspace and electric energy intensity rate at 205 kW h/m 2 (EIA, 2016).
  - Airflow rate through each AHU, *Q*_*a*_ = *C*_*t*_ x *Q*_*b*_, for San Francisco was estimated at 309 cmm (or 18,541 m^3^/h), assuming the base airflow rates, *Q*_*b*_, of 375 cfm/ton (Glawe, 2013).
- Percentage of outside air entering the AHU, *O*_*a*_ was assumed at 100%, consistent with (Licina and Sekhar, 2012).

ACH system components were consistent with RWH system (Table 1); the ACH storage tank was also determined by choosing the minimum of the monthly average collection and monthly average demand (12-month averages). Drain pan was excluded, assuming it was a part of HVAC system. The input amount per functional unit of the ACH component was determined using ACH collection rate, % demand met, and service life.

### 2.4. Life cycle inventory (LCI) for RWH and ACH systems

LCI databases were compiled from Ghimire et al. (2017), ecoinventv. 2.2 (Ecoinvent, 2012), Building for Environmental and Economic Sustainability (BEES) (NIST, 2013), EPA ORD LCI database (Cashman et al., 2014), and the U.S. LCI database (NREL, 2013), in addition to publicly available data and peer-reviewed journal articles. To address variability in design parameters and assumptions, the baseline designs of RWH and ACH systems were modified to nine scenarios, of which three types of systems (baseline RWH system, ACH system, and combined system (RWH + ACH)) were tailored to 4-story and 19-story commercial buildings in San Francisco, CA and Washington, DC. The LCI of piping components of systems in taller buildings (19-floors) were estimated linearly using the LCI of piping in the 4-story building. Pumping energy intensity (i.e., energy use per unit volume of water) was estimated using simple power equation as described by Ghimire et al (2017). The LCI data from Ghimire et al. (2017) was adapted by retrofitting additional treatment trains as best management practices for non-potable use (proposed by the Water Environment & Reuse Foundation (Sharvelle et al., 2017)) and by modifying input amounts according to system size (Eq. 5). A process of pathogen inactivation through ultraviolet (UV) radiation and chlorination, as well as a corrosion inhibitor (Orthophosphate) to reduce corrosion due to high purity with a low mineral content of roof runoff and condensate water, were included. Residual chlorine at a 1.5 mg/L (an average of 0.5–2.5 mg/L), similar to criteria for flushing toilets with graywater in California (National Academies of Sciences, 2016; Sharvelle et al., 2017) was used. To make a standard comparison of water supply from two different sources, i.e., RWH and ACH, we used a functional unit of 1 m^3^ of water produced by RWH or ACH for flushing toilets and urinals. This functional unit also accounted for the annual water demand and the 50-year service life for RWH and ACH systems, which is consistent with Ghimire et al. (2017).

LCI data were normalized to the functional unit of 1 m^3^ water supply by incorporating annual water demand, % demand met, and component service life (Eq. 5).

(5) $$F_{input}\;=\;\frac{M}{k\text{ x }D_{a}\text{ x }T}$$

where *F*_*input*_ = Input amount per functional unit of a component (amount/m^3^) *M* = Amount of a component adapted from Ghimire et al. (2017) (amount and unit vary with component, e.g., the amount (mass) of fiberglass used for fabricating a RWH storage tank is 2335 kg and the distribution CPVC pipe length is 152 m *k =* Fraction of demand met by rainwater/condensate supply *D*_*a*_ *=* Total annual demand for flushing toilets and urinals (m^3^/y) *T =* Service life of a component (year)

As a part of data quality assessment, LCI data calculations and LCA modeling processes were reviewed and a LCI data quality scoring was completed for all foreground processes using the U.S. EPA data quality scheme (Edelen and Ingwersen, 2016). Note that EPA’s data quality system addresses representativeness at the flow level and data completeness at the process level, and it is comprised of a pedigree matrix of key characteristics of data quality: time-related coverage, geographical coverage, technological coverage, precision, completeness, representativeness, consistency, reproducibility, sources of the data, and uncertainty of the information (ISO, 2006b, a). Detailed quality assessment for the background datasets was beyond the scope of current study. Note that the foreground processes were the primary concerns to this analysis as they were compiled directly by the authors linking background processes; the background processes are indirectly aggregated data sets linked to the foreground processes (USEPA, 2018).

### 2.5. LCA modeling

LCA of the RWH and ACH systems was conducted using 11 LCIA categories. Publicly available software (OpenLCA version 1.7.2) was used for all LCA calculations, in conjunction with the Tool for the Reduction and Assessment of Chemical and other Environmental Impacts (TRACI) version 2.1, and life cycle inventory databases (USEPA, 2013; OpenLCA, 2018); a LCA system boundary is defined by Fig. 2. Sensitivity analyses of LCIA of RWH system to the percentage (%) of water demand met by RWH and to the number of occupants in the 4-story building in San Francisco were conducted as the two key, influential design parameters. The demand met varied from 7% to 94% (which corresponds to 25% to 325% of baseline demand met) and the number of occupants varied ± 50% of baseline occupants. Note that the % demand met relates to rainwater collection volume and the occupants relate to water demand.

LCIA results were normalized with respect to maximum impact for the systems. LCIA normalization was performed by scenario (number of floors and locations), in addition to overall normalization of the nine scenarios, with respect to maximum impacts.

## 3. Results and discussion

### 3.1. The RWH and ACH system designs in U.S. commercial buildings

**Fig. 3.**
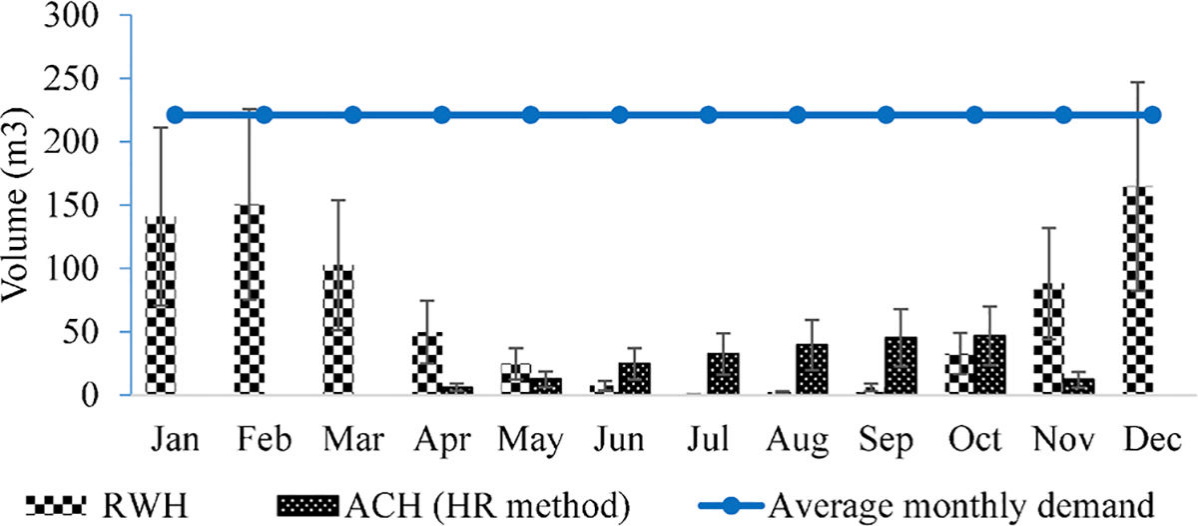
Estimated monthly water demand, and RWH and ACH collection volumes using the humidity ratio (HR) method in a 4-story commercial building in San Francisco, with variation in collections rates of ± 50% as depicted by vertical bars.

RWH collection and demand analysis showed that RWH from the 4-story building with 1000 occupants in San Francisco could supply, on average, 29% of annual demand. RWH collection varied with month, however: the collection volumes were lower during summer months and higher during winter (Fig. 3).

**Fig. A1.**
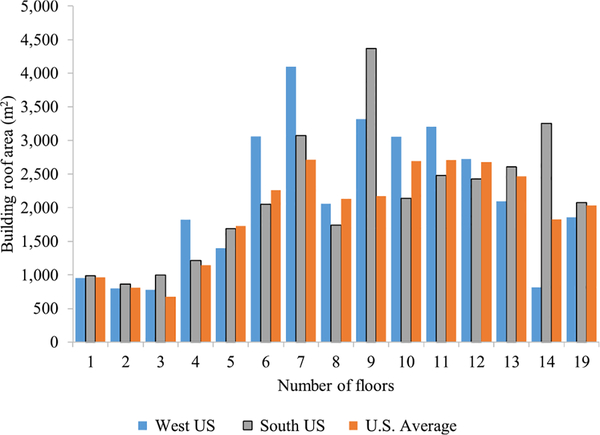
Average roof area of the commercial buildings in West U.S., South U.S., and the U.S. in 2012. The roof area was estimated by dividing the total floorspace per building by number of stories obtained from the Commercial Buildings Energy Consumption Survey (CBECS) of the U.S. Energy Information Administration’s public data (EIA, 2016). Note that the exact number of floors were available up to 14 floors, but 15 to 25 floors and over 25 floors were grouped together to maintain confidentiality of the building respondents. Therefore, the data for the 19-floor was estimated as an average of the group, except for West U.S. that was obtained from Morelli (Personal communication).

**Fig. A2.**
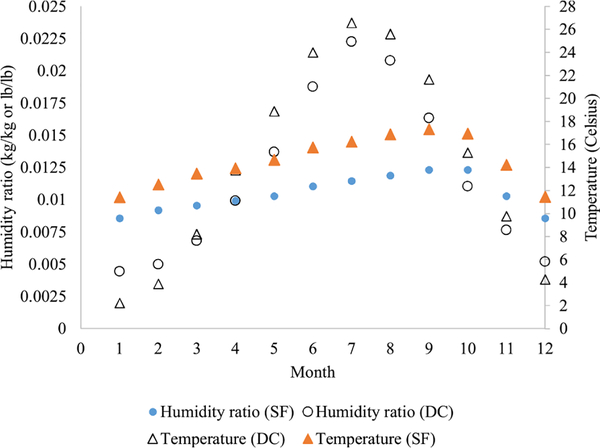
Humidity ratio and temperature in San Francisco (SF) and Washington (DC).

As shown in Fig. 3, because estimated condensate volumes were negative for December through March when they would not be used, they were set to zero. The negative volumes were due to the lower HR of incoming outdoor air than supply air leaving the AHU. Importantly, RWH collections were greater during months when AC condensate collections were lower or not in use, such as January—April. The size of the RWH storage tank for San Francisco was determined to be 17,000 gallons or 64 m^3^ (Table 2). The storage tank size varied by location and the number of floors in a building, since the average roof area and rainfall varied with number of floors and location (Table 2, and Appendix Fig. A1). The size of ACH tank for the San Francisco 4-story building was estimated at 4806 gallons or 18 m^3^. The estimated potential monthly volume of condensate collection from the commercial building in San Francisco ranged from 6 m^3^ in April to 47 m^3^ in October, with the monthly average at 18 m^3^ (Fig. 3, Table 2). A total annual ACH collection volume of 219 m^3^ met 8% of total annual demand, although optimum monthly ACH collection occurred in September and October (Fig. 3). These estimates were greater (ACH storage tank volume was 46 m^3^) for Washington, DC (Fig. 3), which was consistent with humidity ratio and temperature (see Appendix Fig. A2). The variation in RWH and ACH collections, by month, suggested a combined system of RWH and AC condensate to ensure that water demands are met throughout the year and to maximize storage tank usefulness.

**Fig. A3.**
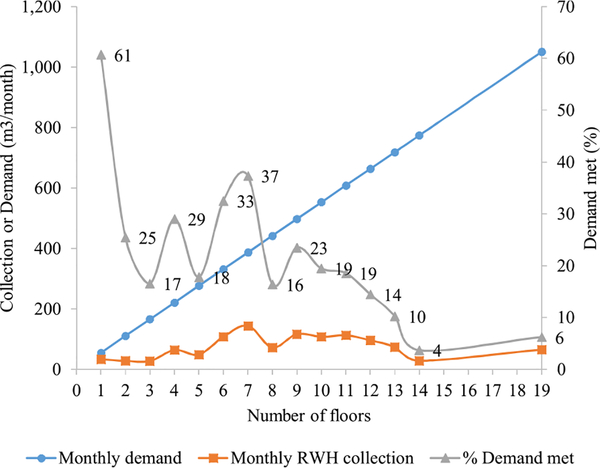
Estimated monthly RWH collection, water demand, and percentage demand met of commercial buildings in San Francisco. Note that the collections and demands varied by months and based on the average monthly collections and demands for each floor category.

**Fig. A4.**
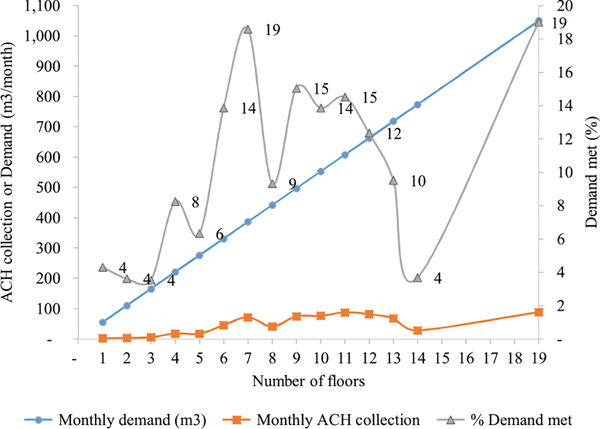
Estimated monthly ACH collection, water demand, and percentage demand met of commercial buildings in San Francisco. Note that the collections and demands varied by months and based on the average monthly collections and demands for each floor category.

The percentage annual demand met by RWH in San Francisco varied from 29% to 6% for 4-story and 19-story buildings, respectively. The demand met by ACH was higher, at 19% for a 19-story building (Table 2). Percentage of demand met varied with the number of floors (Fig. A3 and Fig. A4) and month (Fig. 3). For example, the % annual demand met by ACH was 4% and 19% for 1-floor and 19-story buildings, respectively, in San Francisco, due to the differences in the air conditioned floorspace area relative to occupants’ increasing demands. For these 1-floor and 19-story buildings, corresponding air conditioning floorspace was 952 m^2^ and 35,302 m^2^, while occupants’ demands were 55 m^3^ and 1050 m^3^, respectively (Appendix Figs. A1 and A4). For RWH, rainwater collection varied with roof area of each building category (Fig. A3) since the number of occupants relative to roof area increased with larger buildings, the 1-floor building met the greatest % annual demand (61%) whereas % demand met decreased for larger buildings (6% for 19-story).

### 3.2. Life cycle impact assessment (LCIA)

**Fig. 4.**
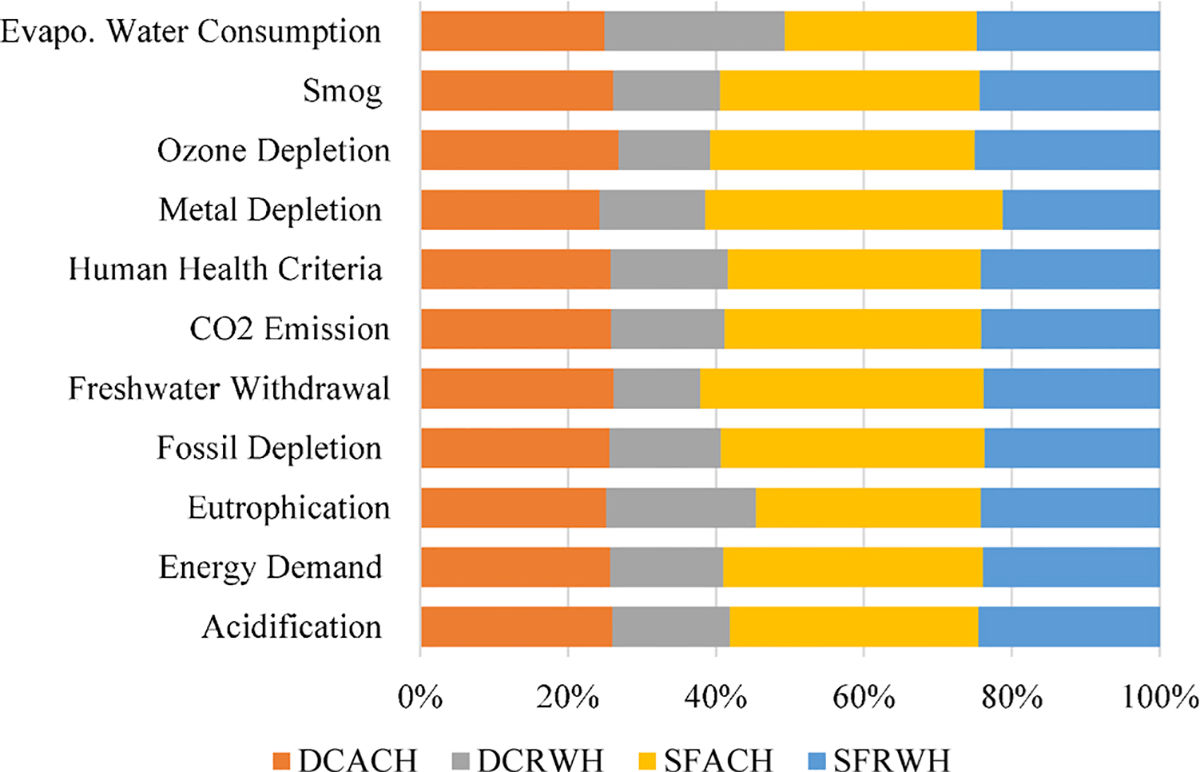
Percentage (%) comparison of LCIA categories of RWH and ACH systems in Washington (DC) and San Francisco (SF). Percentages are calculated with respect to total value of each LCIA category.

This study shed light on a comparative LCIA of nine scenarios of separate and combined RWH and ACH systems in a 4-story building Washington, DC and in 4-story and 19-story buildings in San Francisco, CA. Eleven LCIA category indicators (Acidification, Energy Demand, Eutrophication, CO_2_ Emission, Fossil Depletion, Freshwater Withdrawal, Human Health Criteria, Metal Depletion, Ozone Depletion, Smog, and Evaporative Water Consumption per functional unit of 1 m^3^ of RWH and ACH delivery) were addressed. The % comparison of baseline systems’ LCIA results showed that RWH and ACH systems in 4-story buildings at Washington and San Francisco locations performed equivalently in Evaporative Water Consumption (24–26% of total Evaporative Water Consumption) (Fig. 4).

For the remaining LCIA categories, however, the results varied across the systems. As shown in Fig. 4, the San Francisco ACH system had the largest LCIA results (from 30% Eutrophication to 40% Metal Depletion) than San Francisco RWH (from 21% Metal Depletion to 24% Eutrophication) primarily due to lower ACH collection volume.

**Fig. 5.**
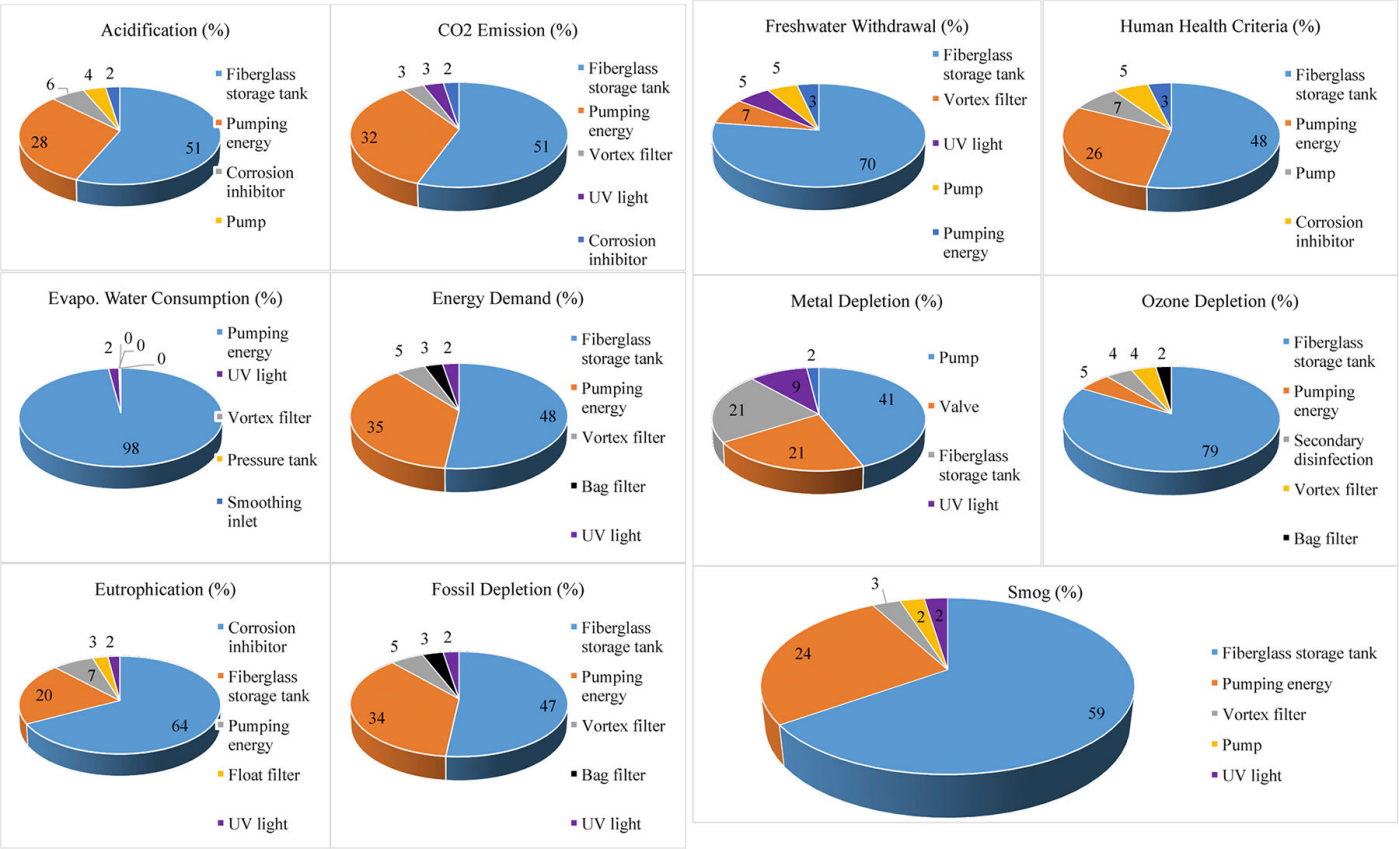
Major (top five) contributing components of baseline RWH system in a 4-story building in San Francisco to selected life cycle impact assessment categories.

The impact equivalency in Evaporative Water Consumption was due to the dominating pumping energy (98%) that was equivalent among systems in 4-story buildings, as shown in Fig. 5; the % contributions of all other components were very small to Evaporative Water Consumption. This component-specific LCIA analysis of the baseline RWH system in San Francisco showed that the storage tank and pumping energy, together, dominated (≥74%) in nine categories except Metal Depletion (41% by pump) and Eutrophication (64% by corrosion inhibitor). Storage tank alone dominated (≥47%) in eight categories, and pumping energy dominated in Evaporative Water Consumption at 98%. The impact contribution of storage tank would be different when ACH and RWH tank size varied with building sizes and locations; more specifically pumping energy’s contribution to total impacts would be even greater in 19-story buildings due to larger pumping energy intensity.

**Fig. B1.**
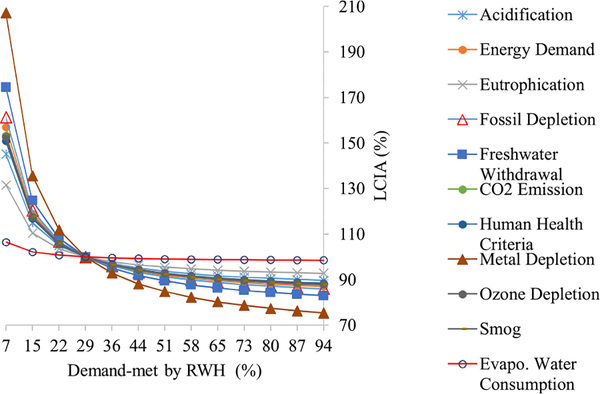
Sensitivity analysis of LCIA categories of RWH systems to the percentage (%) of demand met by RWH in a 4-story building in San Francisco. Percentage (%) values were estimated with respect to baseline RWH system demand met at 29%.

**Fig. B2.**
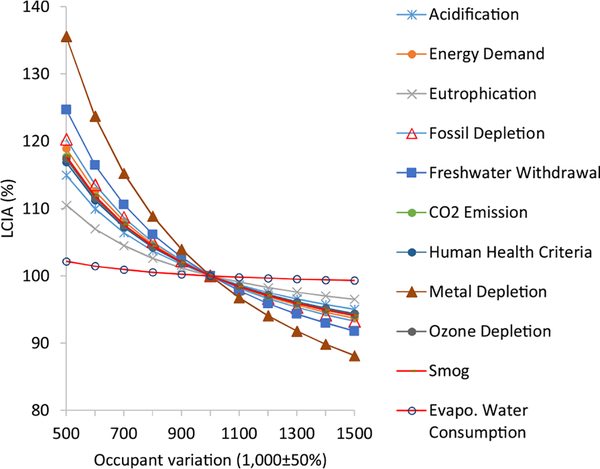
Sensitivity analysis of LCIA categories of RWH systems to number of occupants in a 4-story building in San Francisco. Percentage (%) values were estimated with respect to baseline RWH system building with 1000 occupants.

Sensitivity analysis of LCIA of the baseline RWH system to percentage of water demand met by RWH and to number of occupants in a 4-story building in San Francisco showed impact-specific results (see Figs. B1 and B2, Appendix). Impacts varied inversely with the % demand met and number of occupants because the LCIA results were normalized by water demand.

The greatest variation occurred in Metal Depletion impact — ranging from 75% to 207% for demand met at 94% to 7% (i.e., 325% to 25% of baseline % demand met), respectively, and from 88% to 136% for number of occupants at 1500 to 500 (i.e., 150% to 50% of baseline occupants), respectively — with respect to the baseline RWH system. The variation rate was higher below the baseline value due to lower input amounts normalized by the volumetric water supply.

LCIA analysis of nine scenarios showed impact values of RWH systems in 4-story buildings in San Francisco were lower than ACH system except equivalent in Evaporative Water Consumption; the LCIA values of combined system (RWH + ACH) in San Francisco’s 4-story building were equivalent to RWH’s (Table A2. Appendix). However, the LCIA values of ACH system in San Francisco 19-story building were lower than RWH system except Evaporative Water Consumption, due to the larger volume of ACH collection, and the impacts of combined system were equivalent to the ACH system. Likewise, LCIA values of RWH systems in 4-story buildings in Washington were lower than the ACH system except equivalent in Evaporative Water Consumption; and the impact values of combined systems were equivalent to RWH systems.

**Fig. 6.**
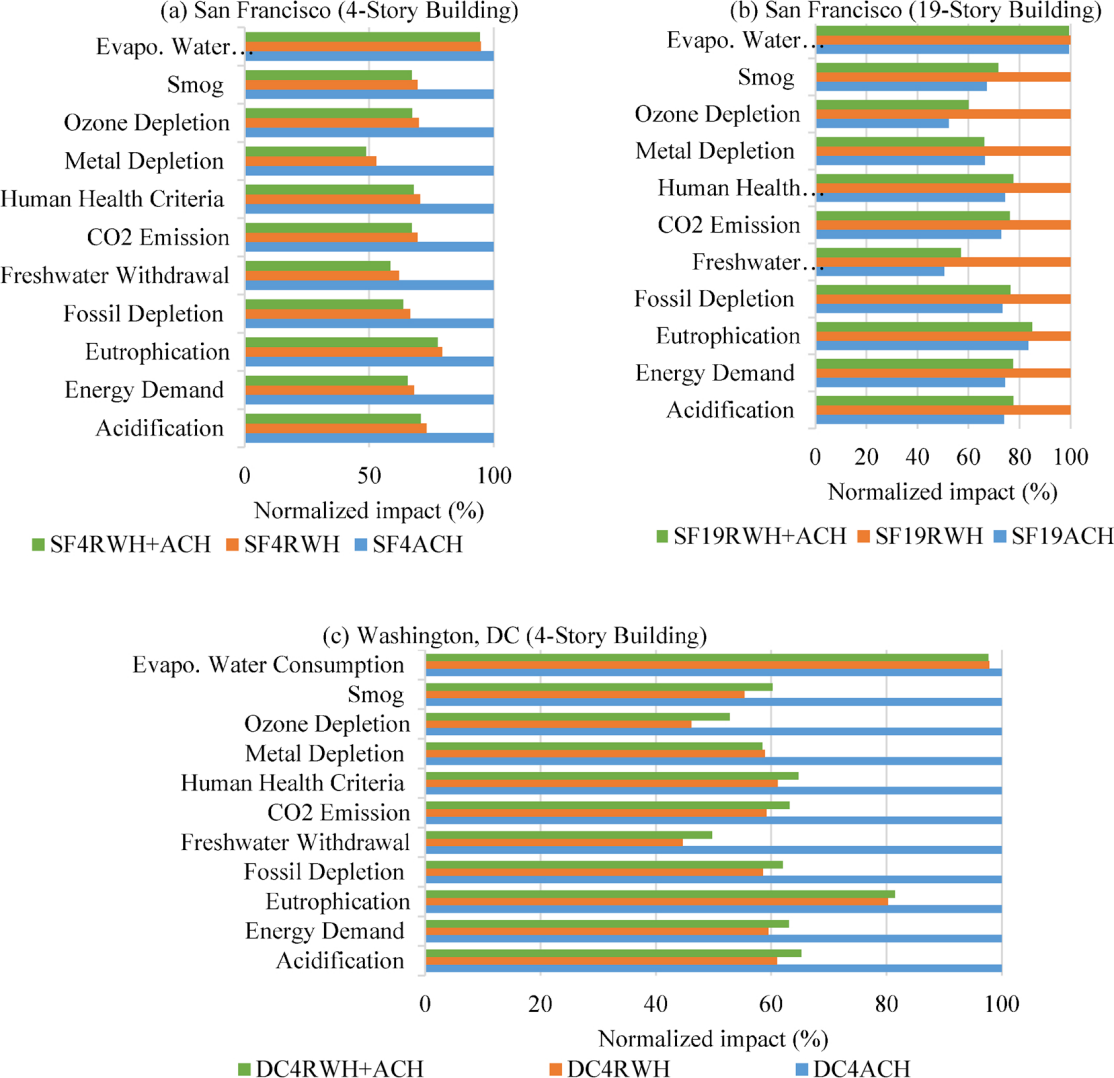
Normalized life cycle impact assessment categories for RWH and ACH systems in: (a) San Francisco 4-story building, (b) San Francisco 19-story building, (c) Washington (DC) 4-story building. Note: SF4RWH = San Francisco or SF 4-story RWH system; SF4ACH = SF 4-story ACH system; SF19RWH = SF 19-story RWH system; SF4RWH+ACH = SF 4-story RWH +ACH system; SF19RWH+ACH = SF 19-story RWH +ACH system; DC4RWH = DC 4-story RWH system; DC4ACH = DC 4-story ACH system; DC4RWH+ACH = DC 4-story RWH +ACH system. Percentage (%) values were estimated with respect to maximum impact values for the scenarios.

To put into perspective, these results were normalized to the maximum impact systems for each location and number of floors (Fig. 6). Normalization of the 11 LCIA categories showed that RWH system in 4-story building in San Francisco outperformed ACH systems (53–79% of ACH impacts) except equivalent in Evaporative Water Consumption. Results showed that the combined (RWH + ACH) system in a 4-story building in San Francisco performed better than a separate ACH system (LCIA at 100%), except in the Evaporative Water Consumption category, and performed similarly to the RWH system in all LCIA categories (Fig. 6a). However, San Francisco’s ACH system in 19-story building outperformed the RWH system (51–83% of RWH impacts) due to the larger volume of ACH collection, except equivalent in Evaporative Water Consumption. The combined system in a 19-story building in San Francisco performed equivalently (57%–85% of RWH impacts) to ACH system (Fig. 6b); as shown in Figure, the equivalent LCIA impacts of the ACH system and combined system in a 19-story building in San Francisco ranged, respectively, from Energy Demand at 74% and 77% to Evaporative Water Consumption at 99% and 99%, with respect to maximum impacts of RWH system at 100%.

Similar to the 4-story building systems in San Francisco, the RWH system in DC outperformed ACH systems (45–80% of ACH impacts) except equivalent in Evaporative Water Consumption. The DC combined system outperformed ACH system in all categories except in the Evaporative Water Consumption category at 98%; however, it performed equivalently to RWH system primarily due to higher volumetric RWH collection. Equivalent LCIA impacts of the DC RWH system and combined system ranged, respectively, from Energy Demand at 60% and 63% to Evaporative Water Consumption at 98% and 98%, with respect to maximum impacts of ACH system at 100% (Fig. 6c). For all three buildings, the LCIA values of combined system were equivalent to the better-performing option (≤4–8% impact difference compared to the maximum system). DC’s RWH system performed better than ACH primarily due to the smaller volume of condensate collection to which impacts were normalized.

**Fig. B3.**
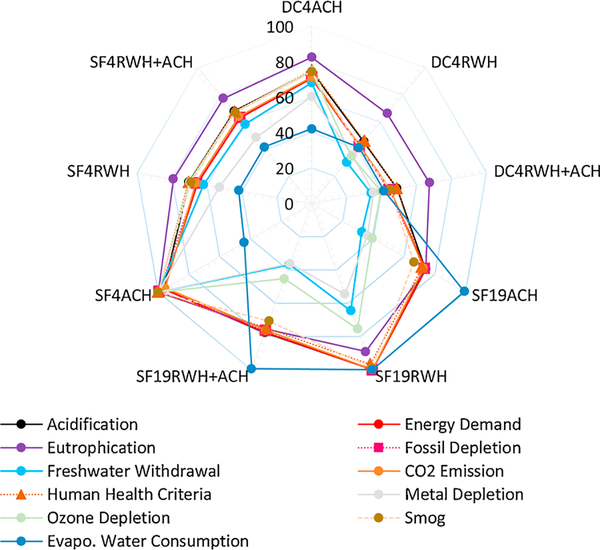
Comparison of the normalized life cycle impact assessment categories (%) of nine scenarios of RWH and ACH systems in San Francisco (SF) and Washington (DC): SF4RWH = SF 4-story RWH system; SF4ACH = SF 4-story ACH system; SF19RWH = SF 19-story RWH system; SF4RWH+ACH = SF 4-story RWH +ACH system; SF19RWH+ACH = SF 19-story RWH +ACH system; DC4RWH = DC 4-story RWH system; DC4ACH = DC 4-story ACH system; DC4RWH+ACH = DC 4-story RWH +ACH system. Percentage (%) values in the axes were estimated with respect to maximum impact values for the scenarios. SF4ACH and SF19RWH scenarios have the maximum values at 100% for different impact categories.

Further, normalization of LCIA of all nine scenarios to the maximum impact systems in each category provided a perspective for comparison of all systems. Results showed that the six scenarios of separate and combined RWH and ACH systems in 4-story buildings in Washington (DC) and San Francisco (SF) (DC4RWH, DC4ACH, DC4RWH + ACH, SF4ACH, SF4RWH, and SF4RWH + ACH, see Fig. B3., Appendix) performed equivalently (41%–44%) in Evaporative Water Consumption impact, due primarily to the dominating pumping energy (98%) that was equivalent among systems in 4-story buildings. The three remaining scenarios of separate and combined RWH and ACH systems in a 19-story building in San Francisco (SF19ACH, SF19RWH, and SF19RWH + ACH) performed worse (≈100%) in Evaporative Water Consumption given their greater pumping requirement. Among the nine scenarios, the San Francisco ACH system in a 4-story building performed worst, with maximum impacts at 100% in six LCIA categories: Human Health Criteria, Metal Depletion, Ozone Depletion, Smog, Freshwater Withdrawal, and Eutrophication due to lower ACH collection volume; the San Francisco RWH system in a 19-story building performed worst with maximum impacts at 100% in the remaining five categories: Acidification, Energy Demand, Fossil Depletion, CO2 Emission, and Evaporative Water Consumption due to greater electric energy intensity rate.

LCIA results of baseline RWH system for Washington, DC were lower than systems in San Francisco primarily due to larger amounts of rainwater collected, i.e., larger value of percentage demand (77%) met. The % demand met by ACH and RWH in San Francisco were lower, at 8.3% and 29%, respectively. While a combined RWH + ACH system in a 4-story building (DC) met 98% of water demand, a combined system in a 4-story building (San Francisco) met only 37%. Contributing pipe length, tank mass intensity (i.e., mass per cubic meter of RWH or condensate collection), and pumping energy intensity (kWh/m^3^) also played roles in impact variations. For taller (e.g., 19-story) buildings, pumping energy increased by 240% (from 0.19 kW h/m^3^ to 0.46 kW h/m^3^) which was the second highest dominating component for most impact categories of baseline RWH system (Fig. 5). The highest dominating component was fiberglass storage tank. The storage tank intensity (kg/m^3^) was lowest for DC4RWH and SF19ACH, which also contributed to lower LCIA values.

It is noted that the LCIA indicator values are influenced by LCIA characterization methods, as well as LCA model parameters and information uncertainty (e.g., information availability, accuracy, or a certain degree of spatial and temporal variation) (Ross et al., 2002; Guo and Murphy, 2012; Lesage et al., 2018). A data quality scoring was completed for all foreground processes using the US EPA data quality scheme (Edelen and Ingwersen, 2016). Scoring of the foreground LCI data quality assessment indicated that the environmental impacts were associated with background data not included in initial data quality scoring. The foreground data could be improved through updating key flows with newer data. The sensitivity of key influential model parameters, % demand met and number of occupants to the LCIA results was addressed; however, the detail scoring of background LCI data quality assessment could be an important next step.

RWH and ACH use options other than toilet flushing, such as drinking, irrigation, ornamental water features, manufacturing processes, and laundry, require different treatment options and system size, depending upon total water supply and demand, which was beyond the scope of our study. Estimating rainwater collection and storage tank size using gross average estimates of rainfall (e.g., monthly) are usually precise enough for design purposes (Glawe, 2013), although daily or hourly rainfall data over an extended period may be used if necessary (Ghimire et al., 2012, 2017). Similarly, a design recommendation related to ACH is to select the appropriate storage tank by analyzing local temperature and humidity ratio, in addition to system operation requirements (e.g., cooling capacity, air flow, conditioning energy intensity, conditioned floorspace, and AHU operation hours). Regulatory requirements governing water use and water quality, as well as life cycle costs and plumbing codes can also influence design and LCIA. However, those are beyond the scope of this study.

## 4. Conclusions and study implications

LCA of ACH and RWH systems for non-potable use in commercial buildings in San Francisco (CA) and Washington (DC) was presented: nine design scenarios were addressed along with sensitivity analyses of demand met and number of occupants. A comparison of systems by number of floors in the 4-story Washington building showed that the combined system performed similar to a separate RWH due to greater collection of the DC RWH. Similar patterns were observed in a 4-story building in San Francisco. A combined (RWH + ACH) system in San Francisco had lower LCIA results than a separate ACH system in a 4story building, except equivalent Evaporative Water Consumption. However, comparing systems in the 19-story San Francisco building showed that the combined system performed equivalently to a separate ACH system or better than a separate RWH system, primarily due to greater collection of AC condensate in the taller building. These results concluded that, the combined system preformed equivalently to the better-performing option (≤4–8% impact difference compared to the maximum system) in terms of LCIA indicators: Acidification, Cumulative Energy Demand, Eutrophication, CO_2_ Emission, Fossil Depletion, Freshwater Withdrawal, Human Health Criteria, Metal Depletion, Ozone Depletion, Smog, and Evaporative Water Consumption per functional unit of 1 m^3^ of RWH and ACH delivery for toilet and urinal flushing for all three buildings in Washington and San Francisco. In other words, combined systems’ LCIA indicators were lower than or equivalent (≤8% difference) to separate systems in both locations. Storage tank size, water collection rates, and pumping energy were key parameters dictating most indicators.

This analysis and the results should be useful for planning RWH and ACH at similar climatic locations. Specifically, societies striving for sustainable and healthy communities can use the results and methods in assessing innovative water reuse systems towards meeting their comprehensive goals of life cycle environmental performance. In different climatic conditions, planners must pay careful attention to system design — with appropriate storage size, pumping calculations, and estimates of annual water collections. An important next step would be to perform LCA of other RWH and ACH use options, such as drinking, irrigation, ornamental water features, manufacturing processes, and laundry, and compare it with conventional municipal water supply systems. Our approach is applicable to such efforts at other locations for which relevant information such as system design parameters and LCI data are acquired. Our transparent set of LCI data and LCA models are generally transferrable in recreating LCA models of RWH and ACH systems at other locations with varying climates, building scales, and number of floors in a building.

Both RWH and ACH systems, individually, have potential to reduce environmental impacts, but a combination of the two resulted in equivalent or lower LCIA values, primarily due to higher total water displaced by combined RWH and ACH. Therefore, it can be said that a combined system should be preferred everywhere; however, site-specific RWH and ACH systems should be designed by analyzing local climate (rainfall) and water demand data of the building’s occupants.

Decentralized green infrastructure practices used in water resource management, such as RWH and ACH, are viable strategies for addressing many sustainability issues in the face of global climate change. The current LCA study offered a comprehensive view of life cycle implications including CO_2_ emission, fossil depletion, metal depletion, ozone depletion, smog, evaporative water consumption, freshwater withdrawal, energy demand, and human health criteria. This study was intended to support implementation decisions related to on-site nonpotable use of RWH and ACH systems in different climates.

## Figures and Tables

**Table 1:** Description of the major components of baseline RWH and ACH systems in San Francisco. Note that the input amount per functional unit were modified from Ghimire et al. (2017) by normalizing by annual water demand, % demand met, and service life, specific to San Francisco.

Main component	Sub-component	Material (unit)	Amount per functional unit, RWH (unit/m^3^)	Amount per functional unit, ACH (unit/m^3^)	Service life (year)
Bag filter and housing	Bag filter	polypropylene (kg)	1.97E-05	6.91E-05	15
	Filter housing	polypropylene (kg)	3.94E-04	1.38E-03	15
Corrosion inhibitor	Sodium tripolyphosphate	sodium tripolyphosphate, at plant (kg)	2.05E-03	2.05E-03	N/A
152 m CPVC distribution pipe	1.5 inch (1.5”) CPVC pipe	HCWD 1.5″ 1 m- CPVC cradle-to-gate (kg/m)	5.20E-05	1.83E-04	50
Pumping energy	Electricity	electricity, at residential user (kWh/m3)	1.90E-01	1.90E-01	N/A
1 hp pump (1 unit)	Pump	primarily stainless steel (kg)	1.56E-03	5.48E-03	15
500 Gallon HDPE Day Tank	HDPE Tank	water supply 8″ 1 m - PE cradle-to-gate pipe, tank equivalent length 181 m	4.70E-03	1.65E-02	50
Fiberglass Storage Tank	Fiberglass (FG) Storage Tank	glass fibre (kg)	6.07E-02	5.99E-02	50
	Two FG Access Riser (36” Diameter 3 ft tall)	glass fibre (kg)	2.95E-03	1.04E-02	50
	Two FG Access Collars (36” Diameter)	glass fibre (kg)	2.95E-03	1.04E-02	50
	Two overflow pipe (8” 2 ft HDPE)	water supply 8” 1 m - PE cradle-to-gate (m)	2.53E-04	8.90E-04	50
Floating filter	Filter assembly	stainless steel (kg)	5.90E-05	2.07E-04	15
	Hose	food grade reinforced plastic hose (kg)	1.97E-04	6.91E-04	15
	Floating ball	polyethylene (kg)	1.97E-05	6.91E-05	15
Ultrasonic Level Transmitter (sensor)	Housing	polypropylene housing (kg)	7.87E-05	2.77E-04	15
20 Gallon Pressure tank (steel)	Inner shell tank	rolled steel (16 gauge) (kg)	3.78E-04	1.33E-03	50
	Diaphragm separating air and water	butyl rubber: synthetic rubber, at plant	2.36E-05	8.30E-05	50
	Polypropylene liner	polypropylene, granulate, at plant	2.36E-05	8.30E-05	50
61 m PVC pipe (leading to Day Tank)	2 PVC pipe	water supply 2″ 1 m - PVC cradle-to-gate (m)	5.20E-05	1.83E-04	50
Secondary disinfection (Chloramines, chlorine	Ammonia	ammonia, partial oxidation, liquid, at plant	4.76E-04	4.76E-04	N/A
and ammonia)	Chlorine, gaseous	chlorine, gaseous, diaphragm cell, at plant	2.00E-03	2.00E-03	N/A
Smoothing inlet (1 unit)	Smoothing inlet	stainless steel (kg)	1.03E-04	3.63E-04	40
Level switch (normally open, float) (1 unit)	Float switch and cable	polypropylene (Housing) (kg)	9.45E-05	3.32E-04	12.5
UV light chamber	Housing	316 L stainless steel (kg)	1.72E-03	6.03E-03	11
	Bulbs	quartz (kg)	1.07E-04	3.77E-04	11
	Quartz sleeves	fused silica (kg)	5.37E-05	1.89E-04	11
Solenoid Valve (Brass) (1 unit)	Valve	brass (kg)	8.42E-05	2.96E-04	7.5
Vortex Filter (1 unit)	Housing	polypropylene (kg)	1.55E-03	5.44E-03	40
	Lid	aluminum (kg)	1.18E-04	4.15E-04	40
	Intermediate ring	stainless steel (kg)	2.36E-04	8.30E-04	40
	Filter insert	stainless steel (kg)	1.33E-04	4.67E-04	40
Rainwater harvest	Water, resources, in water	water, rainwater (m3)	1.00E + 00	0.00E + 00	N/A
Condensate harvest	Water, Resource, in air	Water (kg)	0.00E + 00	1.00E + 03	N/A

**Table 2:** Scenario description of the RWH and ACH systems in 4-story and 19-story commercial buildings in Washington (DC) and San Francisco (SF), with major component parameters: DC4RWH = DC 4-story RWH system; DC4ACH = DC 4-story ACH system; SF4RWH = SF 4-story RWH system; SF4ACH = SF 4-story ACH system; SF19RWH = SF 19-story RWH system; DC4RWH + ACH = DC 4-story RWH + ACH system; SF4RWH + ACH = SF 4-story RWH + ACH system; SF19RWH + ACH = SF 19-story RWH + ACH system.

Parameters	Unit	DC4RWH	DC4 ACH	DC4 RWH + ACH	SF4 RWH	SF4 ACH	SF4 RWH + ACH	SF19 RWH	SF19 ACH	SF19 RWH + ACH
Volumetric water supply	m^3^/y	2,043	551	2,594	770	219	989	786	2,394	3,180
Storage tank mass	kg	2,773	1,678	4,451	2,335	657	2,992	2,371	3,247	5,618
PVC pipe length	m	61	61	122	61	61	122	290	290	580
CPVC pipe length	m	152	152	152	152	152	152	722	722	722
Pumping energy	kWh/m^3^	0.19	0.19	0.19	0.19	0.19	0.19	0.46	0.46	0.46
Storage tank volume	m^3^	76	46	122	64	18	82	65	89	154
% Demand met	%	77	21	98	29	8	37	6	19	25
Storage tank mass intensity	kg/m^3^	68	152	86	152	150	151	151	68	88

## Appendix A

**Table A1:** Monthly average precipitation data (1986–01 to 2015–12) (NCEI, 2018) in San Francisco, California.

Month	Precipitation (mm)
January	103.3
February	110.4
March	75.2
April	36.4
May	18.2
June	5.5
July	0.3
August	1.5
September	4.5
October	24.0
November	64.5
December	120.7

**Table A2:** Life cycle impact assessment category values of RWH and ACH systems in 4-sotry and 19-story commercial buildings in Washington (DC) and San Francisco (SF): DC4RWH = DC 4-story RWH system; DC4ACH = DC 4-story ACH system; SF4RWH = SF 4-story RWH system; SF4ACH = SF 4-story ACH system; SF19RWH = SF 19-story RWH system; DC4RWH+ACH = DC 4-story RWH +ACH system; SF4RWH+ACH = SF 4-story RWH +ACH system; SF19RWH+ACH = SF 19-story RWH +ACH system.

Impact category	Unit	DC4ACH	DC4RWH	DC4RWH + ACH	SF19ACH	SF19RWH	SF19RWH + ACH	SF4ACH	SF4RWH	SF4RWH + ACH
Acidification	kg SO2 eq	2.9E-03	1.8E-03	1.9E-03	2.9E-03	3.9E-03	3.1E-03	3.8E-03	2.8E-03	2.7E-03
Energy Demand	MJ	1.2E + 01	7.0E + 00	7.4E + 00	1.2E + 01	1.7E+01	1.3E + 01	1.6E + 01	1.09E + 01	1.0E + 01
Eutrophication	kg N eq	2.3E-04	1.8E-04	1.8E-04	2.0E-04	2.4E-04	2.1E-04	2.7E-04	2.2E-04	2.1E-04
Fossil Depletion	kg oil eq	2.1E-01	1.3E-01	1.3E-01	2.2E-01	3.0E-01	2.3E-01	3.0E-01	2.0E-01	1.9E-01
Freshwater Withdrawal	m3	1.5E + 00	6.8E-01	7.6E-01	7.3E-01	1.4E+00	8.2E-01	2.2E + 00	1.4E + 00	1.3E + 00
Global Warming	kg CO2 eq	5.8E-01	3.4E-01	3.6E-01	5.9E-01	8.1E-01	6.2E-01	7.7E-01	5.4E-01	5.2E-01
Human Health Criteria	kg PM2.5 eq	2.6E-04	1.6E-04	1.7E-04	2.5E-04	3.4E-04	2.6E-04	3.5E-04	2.5E-04	2.4E-04
Metal Depletion	kg Fe eq	7.6E-02	4.5E-02	4.4E-02	4.6E-02	6.9E-02	4.5E-02	1.3E-01	6.6E-02	6.1E-02
Ozone Depletion	kg CFC11 eq	9.9E-08	4.6E-08	5.2E-08	5.2E-08	1.0E-07	6.0E-08	1.3E-07	9.2E-08	8.9E-08
Smog	kg O3 eq	3.3E-02	1.8E-02	2.0E-02	2.9E-02	4.3E-02	3.1E-02	4.4E-02	3.0E-02	2.9E-02
Evapo. Water Consumption	m3 H2O eq	5.3E-04	5.2E-04	5.1E-04	1.2E-03	1.2E-03	1.2E-03	5.5E-04	5.2E-04	5.2E-04
